# Mindfulness vs. Physiotherapy vs. Medical Therapy: Uncovering the Best Postoperative Recovery Method for Low Back Surgery Patients during the COVID-19 Pandemic—A Single Institution’s Experience

**DOI:** 10.3390/jpm14090917

**Published:** 2024-08-29

**Authors:** Giuseppe La Rocca, Vittorio Orlando, Gianluca Galieri, Edoardo Mazzucchi, Fabrizio Pignotti, Davide Cusumano, Paola Bazzu, Alessandro Olivi, Giovanni Sabatino

**Affiliations:** 1 Institute of Neurosurgery, Fondazione Policlinico Universitario A. Gemelli IRCCS, Catholic University, 00168 Rome, Italy; giuseppe.larocca@policlinicogemelli.it (G.L.R.); gianluca.galieri01@icatt.it (G.G.); alessandro.olivi@policlinicogemelli.it (A.O.); giovanni.sabatino@policlinicogemelli.it (G.S.); 2 Neurosurgical Training Center and Brain Research, Mater Olbia Hospital, 07026 Olbia, Italy; fabrizio.pignotti@materolbia.com; 3 Department of Neurosurgery, IRCCS Regina Elena National Cancer Institute, 00144 Rome, Italy; edoardo.mazzucchi@ifo.it; 4 Department of Neurosurgery, Mater Olbia Hospital, 07026 Olbia, Italy; 5 Unit of Medical Physics, Mater Olbia Hospital, 07026 Olbia, Italy; davide.cusumano@materolbia.com; 6 Clinical Psychology Service, Mater Olbia Hospital, 07026 Olbia, Italy; paola.bazzu@materolbia.com

**Keywords:** mindfulness, physiotherapy, low back pain, COVID-19, lumbar spine surgery

## Abstract

Introduction: This study aimed to evaluate the efficacy of mindfulness therapy compared to traditional physiotherapy and usual care in alleviating postoperative pain and improving functional outcomes in patients undergoing lumbar spine surgery during the COVID-19 pandemic. Methods: Ninety patients undergoing lumbar decompression and fusion (LDF) who presented persistent low back pain after surgery were prospectively followed for one year. They were randomly divided into three groups: mindfulness therapy, physiotherapy, and medical therapy. The primary outcome was the improvement of the Oswestry Disability Index (ODI) score postoperatively and at six months follow-up. Results: Both mindfulness and physiotherapy groups showed significant improvement in ODI scores compared to the control group, with mean variations of 10.6 and 11.6 points, respectively, versus 4.9 points in the control group. There was no significant difference between mindfulness and physiotherapy (*p* = 0.52), but both were superior to medical care (*p* < 0.0001 for physiotherapy and *p* = 0.0007 for mindfulness). Conclusions: This study demonstrated that mindfulness therapy is more effective than usual care in improving postoperative outcomes for patients undergoing lumbar spine surgery. In our cohort, its efficacy was comparable to that of physiotherapy, making it a viable alternative, especially when access to healthcare services is restricted, as seen during the COVID-19 pandemic. Future research should validate the findings of this study and examine the long-term effects on surgical patient populations.

## 1. Introduction

The COVID-19 pandemic presented significant challenges to healthcare systems worldwide, particularly in managing postoperative care for patients undergoing surgical procedures, including lumbar spine surgery [1]. These challenges led to prolonged recovery periods for patients, impacting their ability to resume normal activities, including returning to work.

Lumbar degenerative pathology, particularly lumbar spinal stenosis, is a common condition affecting approximately 103 million people worldwide and 11% of older adults in the US [2]. In this context, effective postoperative care is crucial. Traditional physiotherapy is among the most widely used postoperative treatments, yielding favorable results in the recovery of patients following lumbar spine surgery. This approach typically includes both passive and active physical therapies and often incorporates techniques such as transcutaneous electrical nerve stimulation (TENS), capacitive and resistive electric transfer (TECAR) therapy, laser therapy, as well as ultrasound-guided or fluoroscopy-guided pharmacological injections. These methods have been shown to effectively reduce pain and enhance functional recovery in patients with low back pain, including those recovering from lumbar spine surgery [3,4].

In recent years, mindfulness-based interventions have gained attention as potential adjuncts to traditional therapies. Mindfulness-based stress reduction (MBSR) has been found to be beneficial in managing chronic low back pain, with evidence suggesting improvements in pain intensity and quality of life [5]. For instance, Cherkin et al. reported that mindfulness and Cognitive Behavioral Therapy (CBT) led to greater improvements in disability compared to usual care [6]. However, the applicability of these findings to postoperative settings remains less explored.

This study investigates the efficacy of mindfulness therapy as a postoperative intervention compared to traditional physiotherapy and medical care. We aim to determine which method is most effective in alleviating postoperative pain and improving functional outcomes in patients who have undergone lumbar spine decompression and fusion.

## 2. Materials and Methods

### 2.1. Study Design

Following approval by our local ethical committee (no. 276/2020/CE), we conducted a retrospective review of 697 patients affected by degenerative lumbar spine stenosis (DLSS) with signs of instability who underwent lumbar decompression and fusion (LDF) at Mater Olbia Hospital from January 2020 to December 2023. Ninety patients presented with persistent low-back pain one month after surgery; after this period, they were randomly distributed into three postoperative treatment groups: physiotherapy, mindfulness, and medical therapy. All patients were prospectively followed for at least one year by a team composed of a neurosurgeon and a psychologist.

### 2.2. Data Collection

Psychological and functional disability assessments were conducted for patients eligible for lumbar spine surgery who agreed to participate in the study. Additional data included patient demographics such as age, weight, body mass index (BMI), smoking status, and other risk factors. The Oswestry Disability Index (ODI) [7] scale was administered prior to surgery, one-month follow-up after surgery and at post-treatment (physiotherapy, mindfulness, and medical therapy) follow-up. The Oswestry Disability Index (ODI) is a self-assessment scale used to measure the degree of functional disability in patients with low back pain. The total score ranges from 0 to 100, with the following categories: 0–20% (minimal disability), 21–40% (moderate disability), 41–60% (severe disability), 61–80% (extremely severe disability), and 81–100% (complete disability) [8]. The primary outcome measure was defined as achieving an ODI score lower than 20 at follow-up, indicative of minimal or no disability. The secondary outcome measured the difference in improvements in the ODI and back pain following various post-surgical treatments.

### 2.3. Inclusion and Exclusion Criteria

Inclusion criteria for participation in the study were as follows:

- Radiological diagnosis of DLSS.
- Postoperative low back pain.
- Surgical treatment by LDF.
- Age between 18 and 80 years.

Exclusion criteria included:

- Previous or ongoing treatment for psychiatric disorders.
- Undergoing psychotherapy within the past 2 years.
- Presence of chronic pain due to causes other than degenerative lumbar spine disease.
- Diagnosis of active neoplastic disease.
- Presence of cognitive impairment.

### 2.4. Surgical Treatment

All surgical procedures were performed at the same medical institution by a consistent team of surgeons, using a standardized surgical technique. Each patient underwent lumbar decompression and fusion.

Surgeries employed a percutaneous approach for pedicular screw placement, guided by CT-based navigation. In those procedures, the BrainLab Curve 1.2^®^ navigation system (Brainlab AG^®^, Munich, Germany) was integrated with the AIRO Mobile intraoperative CT scan (Brainlab AG^®^, Munich, Germany). The instrumentation systems utilized were manufactured by NuVasive^®^ (San Diego, CA, USA). Intraoperative Neuro-monitoring (IOM) was facilitated by the Nerve Monitor System (NVM5^®^) from NuVasive^®^, employed in every case [9].

Following screw placement, surgical procedures involved laminectomy with flavectomy and lateral recess decompression [10]. A drainage tube was uniformly placed in all cases and removed the day after surgery upon patient mobilization.

### 2.5. Postoperative Treatment Groups

#### 2.5.1. Mindfulness

Patients in the mindfulness exercise group initially underwent a psychological assessment and participated in face-to-face exercises with a psychologist. Following this, they were consistently supported through daily practice sessions using the ‘Headspace’ mobile application or individual videoconference sessions [11]. This app provided a variety of mindfulness exercises, including guided meditation, deep breathing techniques, body scans, and mindful movement exercises. The comprehensive approach ensured a holistic and tailored experience aimed at promoting relaxation, mental clarity, and emotional well-being.

#### 2.5.2. Physiotherapy

The postoperative physiotherapy group participated in multiple sessions (cycles of ten repeatable sessions) of therapeutic interventions designed first to alleviate pain and muscle tension and then to re-educate and strengthen the muscle tissue. These interventions included antalgic and decontracturating physiotherapy techniques, transcutaneous electrical nerve stimulation (TENS), capacitive and resistive electric transfer (TECAR) therapy, laser therapy and then comprehensive postural re-education and active reinforcing physical therapy, all administered by specialized physiotherapists.

#### 2.5.3. Medical Therapy

Patients in the medical therapy control group who experienced persistent low back pain after surgery were treated with cycles of therapy. Each cycle included a corticosteroid (gradually tapered), a nonsteroidal anti-inflammatory drug (NSAID), a neurointegrative agent, and an analgesic. Additionally, an opioid was provided as needed and medical thermal patches could be used. Each therapy cycle lasted approximately two weeks and could be repeated, if necessary, with a minimum interval of two weeks between consecutive cycles.

Additionally, patients were advised to avoid excessive physical exertion and prioritize rest to facilitate natural healing processes. They received basic support and guidance on maintaining comfort and managing symptoms, such as using over-the-counter pain relief if necessary and following standard postoperative instructions.

### 2.6. Statistical Analysis

The ODI level was measured for each patient at two time points: prior surgery, one month after surgery and six months later, after the end of physiotherapeutic, mindfulness or medical treatment.

For each single patient, the variation in terms of ODI was measured as the difference between the last two time points.

The statistical significance of the difference observed among the three patient groups was calculated using the *t*-test. Differences showing a *p*-value lower than 0.05 were considered statistically significant.

The entire statistical analysis and processing was performed using R software and dedicated packages (R Core Team version, Wien, Austria).

## 3. Results

A total of 697 patients underwent LDF for DLSS between January 2020 and December 2023. Of these, 90 patients who presented with persistent low back pain one month after the procedure were selected and divided into three treatment groups: mindfulness (Group 1), physiotherapy (Group 2), and medical therapy (Group 3). Demographic data are presented in Table 1. Within this cohort, 3 patients were diagnosed with fibromyalgia, 16 with arthrosis, and 15 had previously undergone back surgery. The average BMI of the cohort was 27.64, above the normal range in all groups. Of the 90 participants, 40 were smokers. The mean age of the participants was 59 years (range, 23–75 years).

For surgical and intraoperative characteristics, see Table 2. The intraoperative complications were as follows: cerebrospinal fluid leak in three cases and one screw malposition. The postoperative complications were one subfascial hematoma and one failure of the arthrodesis system.

**Figure 1:**
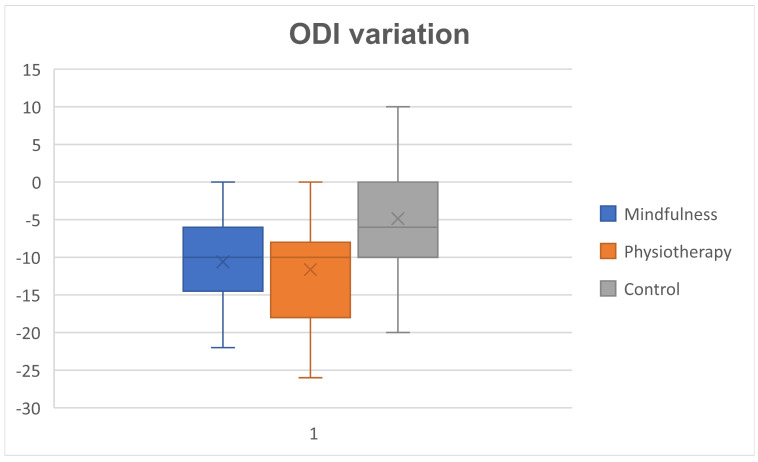
This figure reports the box-plot analysis representing the ODI difference between the right after surgery and post-treatment measurement (six months) in the three patient groups.

Patients undergoing mindfulness or physiotherapy showed a significant improvement in terms of ODI, with mean variations of 10.6 points for mindfulness and 11.6 points for physiotherapy, while the mean variation was 4.9 points for the control group. For each single patient, the variation in terms of ODI was measured as the difference between the two time points, postoperative post-treatment at 6 months and right after surgery (Figure 1).

The difference in terms of ODI variation between mindfulness and physiotherapy was not significant (*p* = 0.52 using *t*-test). A significant gain was observed in comparison to the control group (*p* = 0.0001 for physiotherapy and *p* = 0.0007 with mindfulness). No correlation was found between age or BMI and ODI improvement in the three groups. Subpopulations of patients with fibromyalgia, arthrosis and previous back surgery were too small to establish any statistically relevant correlation.

## 4. Discussion

Mindfulness is a meditation practice rooted in Buddhist principles but free from religious elements. It involves sitting with closed eyes, either cross-legged on a cushion or seated on a chair with a straight back and focusing on the breath. Practitioners may concentrate on the movement of the abdomen while breathing or the sensation of the breath entering and exiting through the nose. When distractions arise, they are acknowledged without judgment, and attention is gently brought back to the breath.

This practice trains individuals to focus on the present moment non-judgmentally. Various psychological treatment protocols incorporating this technique have been developed and validated in clinical settings, demonstrating significant benefits for psychological disorders. Additionally, it has been shown to improve blood parameters related to inflammatory diseases [12]. Practitioners often report enhanced physical and mental well-being and increased creativity. Moreover, this kind of meditation has gained recognition in healthcare for its benefits in pain management and overall well-being; it has been shown that such approaches are associated with reduced pain perception, improved pain coping strategies, and enhanced psychological resilience [13].

The mechanisms by which mindfulness exerts its effects are thought to include the modulation of pain through cognitive and emotional appraisal processes, as well as the reduction in stress and improvement of mood [14].

Several studies have shown moderate-quality evidence that mindfulness-based stress reduction (MBSR) is an effective treatment for chronic low back pain [15,16,17,18]. Petrucci et al. (2021) corroborated these results through a systematic review and meta-analysis. Their study demonstrated that CBT and MBSR significantly reduce pain intensity and enhance quality of life when each one is compared to usual care [19].

A randomized controlled trial (RCT) conducted on 342 patients, as reported by Cherkin et al. (2016) in JAMA, demonstrated that mindfulness-based stress reduction (MBSR) and cognitive-behavioral therapy (CBT) yielded better results in managing back pain and functional limitations in adults with chronic low back pain compared to usual care. The percentage of participants with clinically meaningful improvement on the Roland–Morris Disability Questionnaire (RDQ) was higher for those who received MBSR (60.5%) and CBT (57.7%) than for those who received usual care (44.1%) The improvements from MBSR were found to be comparable to those provided by CBT, a well-established treatment for chronic pain [6,20].

This body of evidence suggests that mindfulness could be a viable alternative to conventional treatments for low back pain, such as medication and physical therapy. The advantages of using this approach include its cost-effectiveness for public health, as shown in a study by Herman et al. (2017) [21], and its broad availability, which can enhance patient compliance with the therapy. Additionally, mindfulness-based interventions have minimal side effects and can be easily integrated into patients’ daily routines, making them a sustainable long-term solution for managing chronic pain.

Despite these promising findings, the literature has several limitations, including heterogeneity in study design, variability in the delivery and duration of mindfulness interventions, and differences in patient populations. Furthermore, there is a lack of studies demonstrating the effectiveness of mindfulness as a therapy for patients undergoing lumbar spine surgery.

Some research has illustrated the potential of mindfulness-based therapy to enhance outcomes for patients undergoing lumbar spine surgery. For instance, the pilot study by Yi et al. (2019) in World Neurosurgery assessed the impact of preoperative MBSR on postoperative outcomes in patients undergoing lumbar spine surgery for degenerative disease. The findings suggested that MBSR might reduce postoperative pain, disability, and opioid use, indicating its potential as an adjunct to standard postoperative care [22]. Moreover, Chavez et al. conducted a study involving 24 patients treated surgically for lumbar spine degenerative disease, with follow-ups at 3 and 12 months. Also, the results of this study suggested that preoperative mindfulness-based stress reduction training might confer benefits in postoperative pain management [23]. Another randomized clinical trial conducted on 25 patients with failed back surgery syndrome (FBSS) showed that MBSR can be a useful clinical intervention for these patients, improving pain perception, quality of life and functional limitation [24].

In our study, we compared the efficacy of mindfulness therapy, physiotherapy, and medical therapy in improving postoperative pain and functional outcomes in patients with DLSS undergoing LDF at a single center during the COVID-19 pandemic. During this period, significant restrictions limited access to healthcare, making it difficult for patients to comply with physiotherapy treatment. To address this challenge, we sought a viable therapy that was affordable, user-friendly, and accessible from home.

Additionally, in previous research we highlighted the pivotal role of psychosomatic traits and psychopathological symptoms in pain perception in patients who underwent spine surgery, emphasizing the importance of targeting these factors in treatment [25]. We found an effective solution in implementing a MBSR program through a smartphone application (Headspace). This approach allowed patients to practice mindfulness therapy from the comfort of their homes without needing to travel. The smartphone app provided a convenient and flexible way for patients to engage in therapeutic practices, ensuring continuity of care and potentially enhancing recovery outcomes despite the constraints imposed by the pandemic.

Out of 697 patients with DLSS who underwent LDF, 90 experienced persistent back pain after surgery. These patients were randomly assigned to three equal groups and followed up for one year. Demographic data were similar in the three groups, with an average BMI of 27.64 and a mean age of 59.

Both mindfulness and physiotherapy groups demonstrated significant improvements in ODI scores—10.6 and 11.6 points, respectively—compared to a 4.9-point improvement in the usual care group. No significant difference was found between mindfulness and physiotherapy, indicating both interventions were similarly effective. These findings highlight the value of integrating mindfulness therapy and physiotherapy into postoperative care protocols, offering significant benefits in enhancing recovery and functional outcomes over usual care, and providing flexible, non-pharmacological treatment options for patients. While physiotherapy remains the most effective strategy for managing postoperative pain, mindfulness therapy serves as a valuable alternative when access to physiotherapy is limited. Additionally, mindfulness-based interventions, particularly through smartphone applications, provide a convenient and accessible means for patients to engage in therapeutic practices. This is especially beneficial in locations or during times, such as the COVID-19 pandemic, where traditional healthcare services are restricted.

This study has limitations, including the number of patients enrolled and being a single-center study. Moreover, the long-term sustainability of the benefits of mindfulness practice for LBP requires further investigation.

## 5. Conclusions

Integrating mindfulness therapy into postoperative care for patients undergoing back surgery could have an effectiveness comparable to that of physiotherapy in improving pain and functional outcomes. This non-pharmacological approach offers a flexible and accessible alternative therapy, especially valuable during healthcare restrictions like those experienced during the COVID-19 pandemic. Future research should focus on validating these findings through larger, multicentric trials to confirm the efficacy of mindfulness therapy across diverse populations and settings. Additionally, exploring the long-term effects of mindfulness and physiotherapy on functional recovery and quality of life in postoperative patients will be essential. Investigating the integration of mindfulness into routine postoperative care, especially in contexts where access to traditional physiotherapy may be limited, could provide valuable insights. Further studies could also examine the cost-effectiveness of these interventions and their potential to reduce reliance on pharmacological treatments, contributing to more sustainable healthcare practices.

## Figures and Tables

**Table 1:** Demographic and clinical characteristics of the study cohort.

Variable	Group 1 (n = 30)	Group 2 (n = 30)	Group 3 (n = 30)	Total (n = 90)
Age (mean), y	58	60	60	59
Sex (M:F)	13:17	17:13	17:13	47:43
BMI (mean), kg/m^2^	27.57	29.15	26.21	27.64
Smoker, no.	14	14	12	40
Previous Back Surgery, No.	3	8	4	15
Arthrosis, No.	4	6	6	16
Fibromyalgia, No.	2	0	1	3

**Table 2:** Surgical characteristics of the study cohort.

Level	Group 1 (n = 30)	Group 2 (n = 30)	Group 3 (n = 30)	Total (n = 90)
L3L4	2	3	2	7
L4L5	12	11	8	31
L5S1	3	6	8	17
L2L4	0	1	1	2
L3L5	9	3	6	18
L4S1	3	3	3	9
L2L5	0	3	1	4
L3S1	1	0	1	2
Average wound size (cm)	8.7 ± 1.1	8.4 ± 1.6	9.2 ± 1.1	8.8 ± 1.3
Mean surgical time (minutes)	128 ± 31	136 ± 42	125 ± 33	133 ± 35
Average Hospital Stay (days)	2.4 ± 0.5	2.3 ± 0.4	2.4 ± 0.9	2.4 ± 0.6
Intraoperative complication	0	3	1	4
Postoperative complication	1	0	1	2

## Data Availability

The data presented in this study are available on request from the corresponding author.
